# Allyl isothiocyanate induces replication-associated DNA damage response in NSCLC cells and sensitizes to ionizing radiation

**DOI:** 10.18632/oncotarget.3026

**Published:** 2015-01-27

**Authors:** Kaushlendra Tripathi, Usama K. Hussein, Roja Anupalli, Reagan Barnett, Lavanya Bachaboina, Jennifer Scalici, Rodney P. Rocconi, Laurie B. Owen, Gary A. Piazza, Komaraiah Palle

**Affiliations:** ^1^ Department of Oncologic Sciences, Mitchell Cancer Institute, University of South Alabama, Mobile, Alabama, USA; ^2^ Faculty of Science, Beni Suef University, Beni Suef, Egypt; ^3^ Department of Genetics, Osmania University, Hyderabad, India

**Keywords:** Dietary isothiocyanates, replication stress, DNA damage response, radiosensitization, non-small cell lung cancer

## Abstract

Allyl isothiocyanate (AITC), a constituent of many cruciferous vegetables exhibits significant anticancer activities in many cancer models. Our studies provide novel insights into AITC-induced anticancer mechanisms in human A549 and H1299 non-small cell lung cancer (NSCLC) cells. AITC exposure induced replication stress in NSCLC cells as evidenced by γH2AX and FANCD2 foci, ATM/ATR-mediated checkpoint responses and S and G2/M cell cycle arrest. Furthermore, AITC-induced FANCD2 foci displayed co-localization with BrdU foci, indicating stalled or collapsed replication forks in these cells. Although PITC (phenyl isothiocyanate) exhibited concentration-dependent cytotoxic effects, treatment was less effective compared to AITC. Previously, agents that induce cell cycle arrest in S and G2/M phases were shown to sensitize tumor cells to radiation. Similar to these observations, combination therapy involving AITC followed by radiation treatment exhibited increased DDR and cell killing in NSCLC cells compared to single agent treatment. Combination index (CI) analysis revealed synergistic effects at multiple doses of AITC and radiation, resulting in CI values of less than 0.7 at Fa of 0.5 (50% reduction in survival). Collectively, these studies identify an important anticancer mechanism displayed by AITC, and suggest that the combination of AITC and radiation could be an effective therapy for NSCLC.

## INTRODUCTION

Lung cancer represents a leading cause of cancer-related mortalities worldwide. More than 200,000 lung cancer cases are diagnosed each year in the United States alone that accounts for over 160,000 deaths [1]. Approximately 85% of the lung cancer cases are classified as non-small cell lung cancer (NSCLC), which is closely linked to smoking or exposure to environmental carcinogens (ex. asbestos, radon, industrial chemicals, etc.) [2, 1]. Regardless of the advances in knowledge of pulmonary carcinogenesis and the development of novel targeted therapies, the overall 5-year survival rate of NSCLC patients is only 15%. According to the data from National Lung Cancer Trial (NLCT) and Surveillance, Epidemiology and End Results (SSER), early detection of NSCLC significantly improves overall survival rate. However, the mortality rate for early diagnosed patients (stage I & II) is still more than 50%. A significant advance in the treatment of NSCLC is molecular profiling based classification of tumor types and direct use of molecularly targeted therapies [3–6]. Unfortunately, targeted therapies are only beneficial in a subset of patients and their long-term use often leads to resistance and recurrence of advanced disease [7, 8]. While it is important to develop more advanced early detection methods, prevention of NSCLC in high-risk individuals and development of new and effective therapeutic strategies is a high priority for public health [9].

Although radiation therapy remains an important treatment regimen for local control of the early-stage, as well as advanced NSCLC, the response rate to radiation among NSCLC patients is highly variable and in the majority of cases, the tumor spreads to distant tissues [7–9]. Though existing guidelines suggest that chemotherapy in combination with radiation can improve survival rates, most patients either do not qualify for this modality or need to go on salvage therapy that requires additional chemotherapy or radiation, which compromises their quality of life and overall survival. Moreover, this variance is also observed in clinical toxicity data of NSCLC patients experiencing treatment related toxicities and effects. Therefore, considering the heterogeneity of response to radiation and chemotherapy, as well as their related toxicities, the development of methods and therapeutic strategies that predict clinical outcome of the disease would significantly benefit the patients [9]. An approach to solve this problem is the use of agents that exhibit tumor specific cytotoxicities that potentiate radiation-induced cell death [9].

Dietary isothiocyanates (ITCs), such as allyl isothicyanate (AITC), phenethyl isothiocyanate (PEITC) and sulfurophane (SFN) have been well studied as chemopreventive agents in animal models of several cancers, including lung cancer [10–14]. Data from epidemiological studies suggest that the consumption of cruciferous vegetables may lower the overall incidence of cancer. These studies also suggest that a diet rich in ITCs can lower the incidence of lung cancer in current smokers [12, 15, 16]. The mode of action for the chemopreventive activity of dietary ITCs is mainly attributed to detoxification of carcinogens through activation of nuclear factor erythroid-related factor2 (Nrf2), which triggers the expression of phase II enzymes [17, 18]. However, it is obvious from several recent studies that carcinogen detoxification through phase II enzymes may not be the only mechanism by which these compounds prevent cancer. For example, feeding of ITCs several weeks after the exposure to carcinogen prevented tumor initiation in murine models [16]. Likewise, administration of ITCs markedly decreased tumor incidence in animal models that spontaneously develop tumors, in which no external carcinogen is involved [17, 18, 19]. Moreover, numerous studies in human tumor xenograft models and tumor cell lines demonstrated tumor-specific growth inhibitory properties for ITCs [20, 21]. Increasing evidence is also available for their ability to cause cell cycle arrest, induce apoptosis, suppress IκB and Nf-κB (nuclear factor kappa-light-chain-enhancer of activated B cells) signaling and binding to thiol-reactive groups of several cellular targets such as DNA topoisomerase 2, p53 and tubulins [18, 21, 22, 23]. These studies strongly advocate for the existence of additional mechanisms that are independent from carcinogen detoxification for their cancer preventive properties.

The above observations suggest that ITCs may have several cellular targets in proliferating tumor cells and their interference could induce DNA damage and cell cycle arrest. In this study we studied the cell cycle checkpoint and DNA damage response (DDR) and repair pathways elicited by the dietary ITC, allyl isothiocyanate and compared these responses with PITC (phenyl isothiocyanate), a synthetic ITC. These studies demonstrated that AITC induces replication-associated DNA damage and affects cells cycle progression through S-phase that lead to G2 accumulation. Further evaluation of combination therapy with radiation revealed that AITC could be a radiation sensitizing agent and this combination demonstrates synergistic therapeutic activity against NSCLC cells.

## RESULTS

### AITC and PITC exhibits chemotherapeutic activities against NSCLC cells

**Figure 1:**
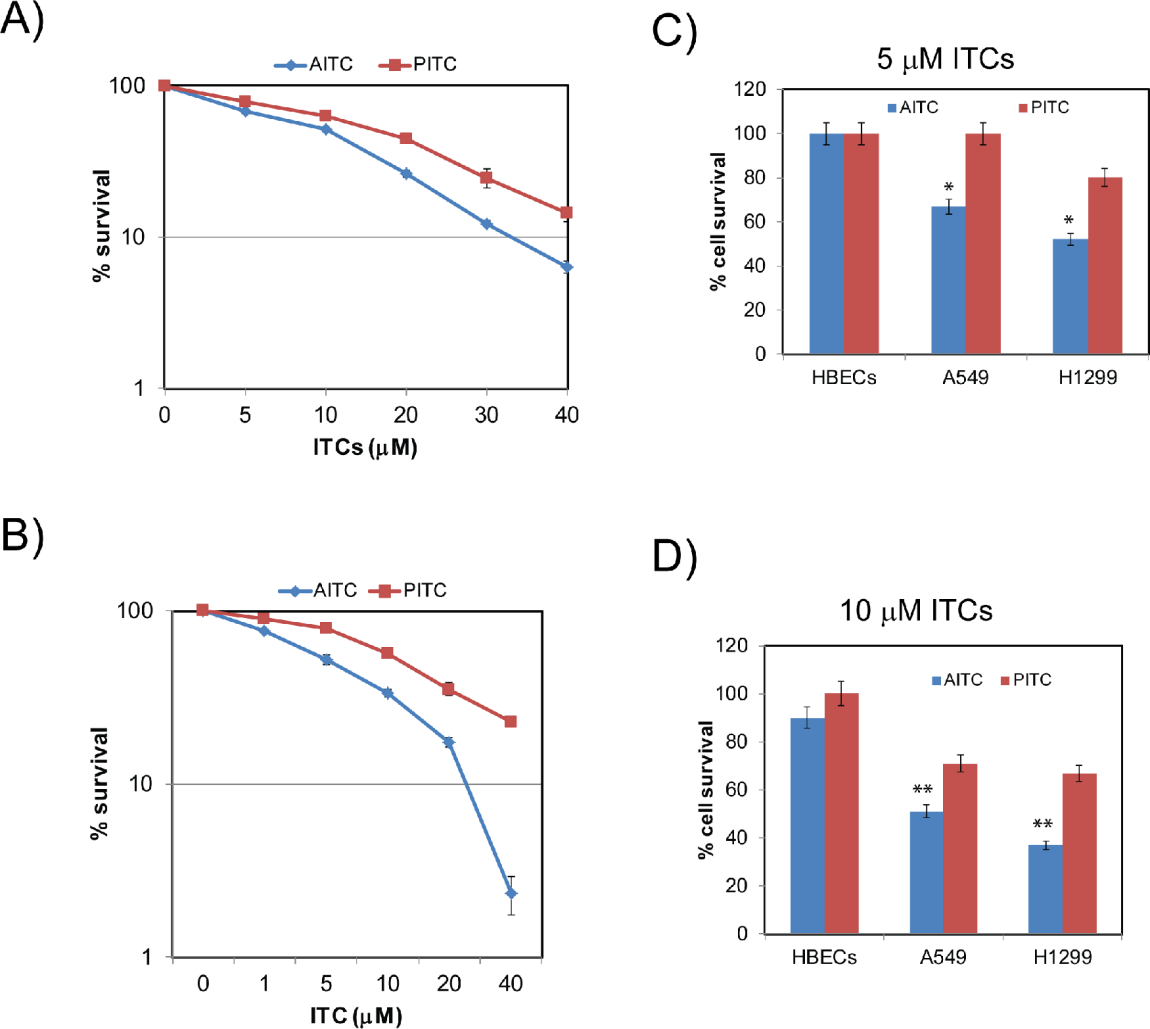
AITC and PITC exhibit cytotoxic effects to NSCLC cells Clonogenic survival assays show AITC and PITC inhibits survival of A549 **(A)** and H1299 **(B)** cells in a dose dependent manner. Cytotoxic effects of AITC and PITC are specific to NSCLC cells at the concentrations of 5 and 10 μM (C and D respectively). Cells were exposed to the indicated concentrations of ITCs for three days and cell viability was assessed using Tryphan blue exclusion assay. Data presented are an average of triplicates and ± SD presented as error bars (*P < 0.01, **P < 0.001).

To assess the antineoplastic activities of dietary isothiocyanate AITC and synthetic isothiocyanate PITC against human NSCLC cells, A549 and H1299 lung tumor cells were exposed to different concentrations of the ITCs and their effects on tumor cell growth were measured by clonogenic survival assays. Clonogenic potential of the NSCLC cell lines, A549 and H1299 were significantly affected by both AITC and PITC in a concentration-dependent manner (Figures 1A and 1B). The IC_50_ values for AITC and PITC were 10 and 15 μM against A549 cells and 5 and 7.5 μM for H1299 cells, respectively. When compared, AITC exhibited superior growth inhibitory properties than PITC in both the NSCLC cell lines. Between the two NSCLC cell lines used, H1299 cells were more sensitive to ITCs compared to A549 cells. These inherent differences in sensitivities between the cell lines may be attributed to their genetic (ex. p53 status) and epigenetic profiles. Moreover, ITCs were shown to exhibit their cytotoxic effects selectively towards tumor cells in several cancer models [23, 24]. Consistent with these studies, both the ITCs exhibited growth inhibitory effects selectively towards cancer cells when compared with normal human bronchial epithelial cells (HBECs). Similar to the clonogenic survival data (Figure 1A and 1B), AITC exhibited significant growth inhibitory affects at 5 and 10 μM concentrations to both the NSCLC cells (Figure 1C and 1D). Although PITC affects were minimal at 5 μM concentration, it exhibited significant growth inhibitory properties at 10 μM concentration to both A549 (Figure 1C) and H1299 (Figure 1C) cells. However, toxic effects of the ITCs used were minimal towards HBECs even at 10 μM concentration (Figure 1D), suggesting their selectivity towards tumor cells.

### AITC treatment slows S-phase progression and induces G2/M cell cycle arrest in NSCLC cells

**Figure 2:**
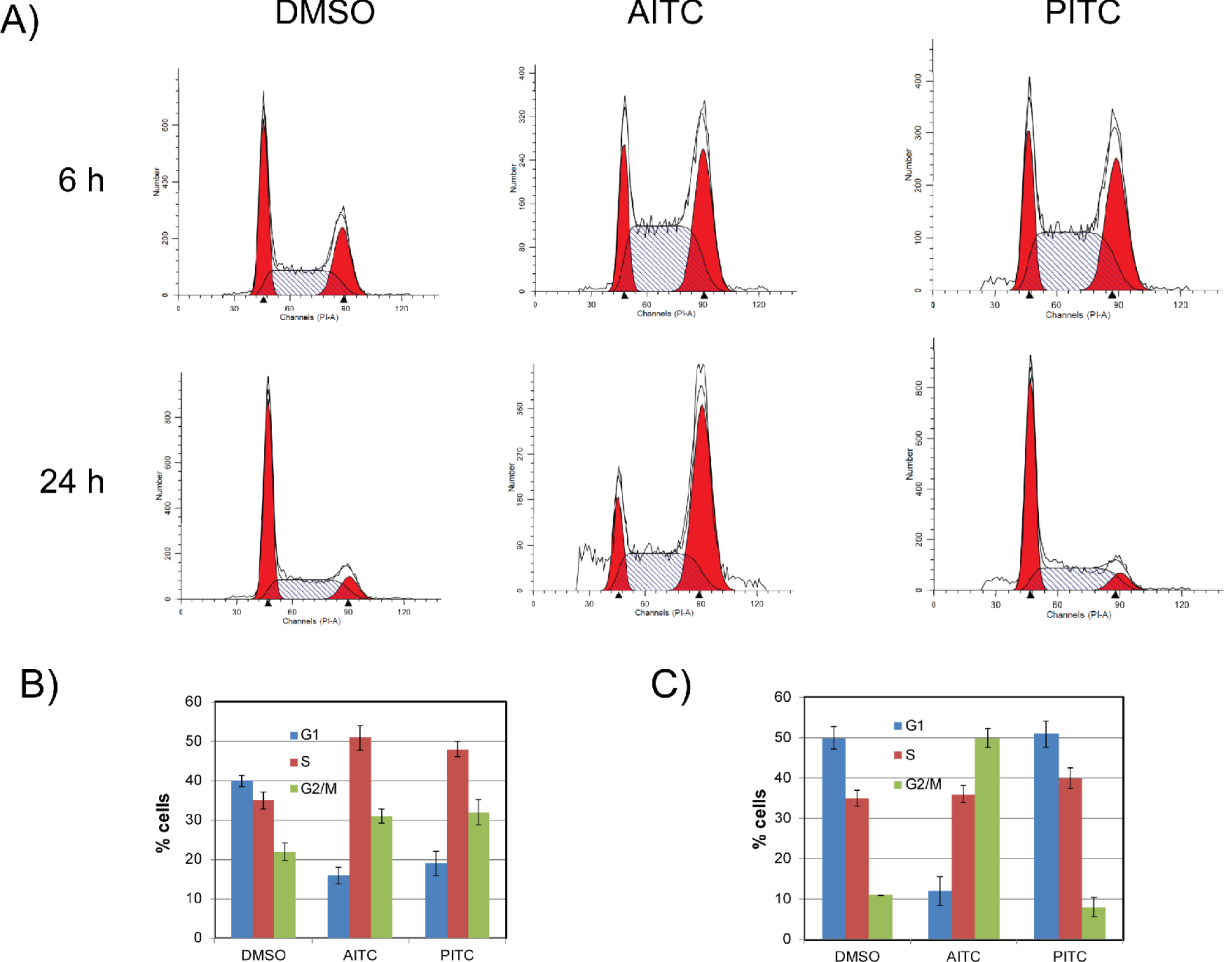
AITC-induces slow progression through S-phase and leads to G2/M arrest H1299 cells were exposed to 20 μM AITC or PITC for 6 (top panel) and 24 hours (bottom panel) and cell cycle profiles were assessed by flow cytometry (A). Data presented in (B) and (C) are average values from three independent experiments for 6 and 24 hours respectively. The error bars presents ± SD.

To gain further insight into the mechanism of their anti-proliferative activities, H1299 cells were treated with either AITC or PITC (20 μM) and their effect on cell cycle progression and distributions were assessed at 6 and 24 hours post-treatment. Exposure of NSCLC cells to AITC and PITC attenuated cell cycle progression through S-phase, as indicated by increased accumulation of cells in S-phase within 6 hours when compared to DMSO (*Dimethyl sulfoxide*) treated cells (Figure 2A, top panel and Figure 2B). However, longer time incubation (24 hours) exhibited differential responses. As shown in the Figures (Figure 2A, bottom panel and Figure 2C), at 24 hour time point, AITC treated cells accumulated in G2/M phases, where as PITC treated cells recovered from transient cell cycle arrest at this concentration. A similar pattern of cell cycle distribution was observed in A549 cells (Figure S1A). These variations in the cell cycle distribution for AITC and PITC indicate either they have differential binding affinities to their targets or they may have different cellular targets. However, their ability to inhibit S-phase progression indicates that ITCs may interfere with DNA replication directly or they may induce replication-associated DNA damage, which in turn can activate checkpoint responses to inhibit the progression of cell cycle.

### AITC exhibits chemotherapeutic activities to NSCLC cells by inducing replication-associated DDR

**Figure 3:**
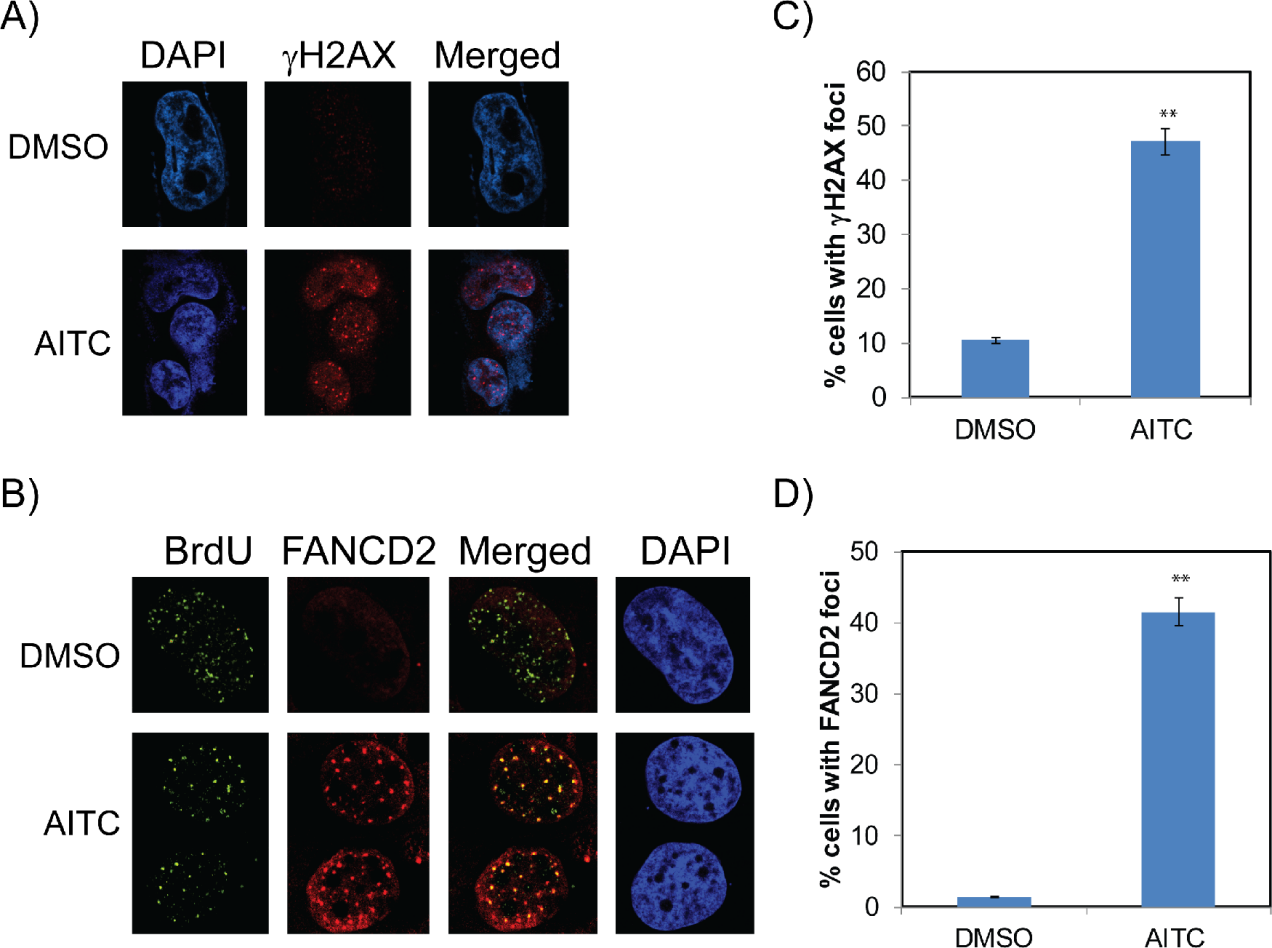
AITC-induces replication-stress mediated DDR in NSCLC cells H1299 cells were treated with 20 μM AITC or DMSO for six hours and assessed for formation of γH2AX **(A)** and FANCD2 foci **(B)**. Number of cells positive for γH2AX foci **(C)** and FANCD2 foci **(D)** were presented in histograms. To assess the co-localization of FANCD2 foci with replication forks, cells were labeled with BrdU for 20 min. At least individual 10 fields were counted and ± SD presented as error bars (**P < 0.001).

To further assess if ITCs mediated cell cycle arrest is due to the replication mediated DDR, A549 and H1299 cells were exposed to AITC and PITC and assessed for the replication stress-mediated DDR proteins by immunofluorescence microscopy. As shown in Figure 3, exposure of H1299 cells to AITC for 6 hours induced a robust increase in γH2AX (phosphorylated form of histone 2 variant X at Serine 139) foci (Figure 3A) and FANCD2 (Fanconi anemia, complementation group D2) foci (Figure 3B) compared to DMSO treated cells. AITC treated cells exhibited over a 4 and 3 fold increase in γH2AX foci and FANCD2 foci positive cells, respectively (Figures 3C and 3D). Similar results were observed in A549 cells treated with AITC (Figures S3A and S3B). It is well known that Fanconi anemia (FA) DNA repair pathway proteins associates with replication machinery and forms foci at the stalled or collapsed replication forks [25, 26]. To assess this, we transiently labeled the cells with BrdU and assessed the localization of BrdU and FANCD2 foci. As expected, DMSO treated cells showed few FANCD2 foci positive cells and most of the cells exhibited pan nuclear staining (Figure 3B). Consistent with γH2AX foci formation, cells exposed to AITC induced robust FANCD2 foci formation. Interestingly, most of the FANCD2 foci formed in AITC treated cells exhibited co-localized with BrdU foci (Figure 3B), indicating stalled or collapsed replication forks in these cells. Additionally, as revealed by the flow cytometry analysis, ITCs treated cells exhibited increased number of EdU positive cells at 6 hours' time point, indicating transient accumulation of cells in S-phase (Figure S5). Together these results indicate that exposure of NSCLC cells to AITC induces replication stress-associated DDR, which slows cell cycle progression though S-phase and accumulates them in G2/M phases. Furthermore, AITC-induced cytotoxicity was dramatically reduced when NSCLC cells were pretreated with aphidicolin, an inhibitor of DNA replication (Figure S4A and S4B). These results further suggest that AITC-induced cytotoxicity is at least partly dependent on active replication.

**Figure 4:**
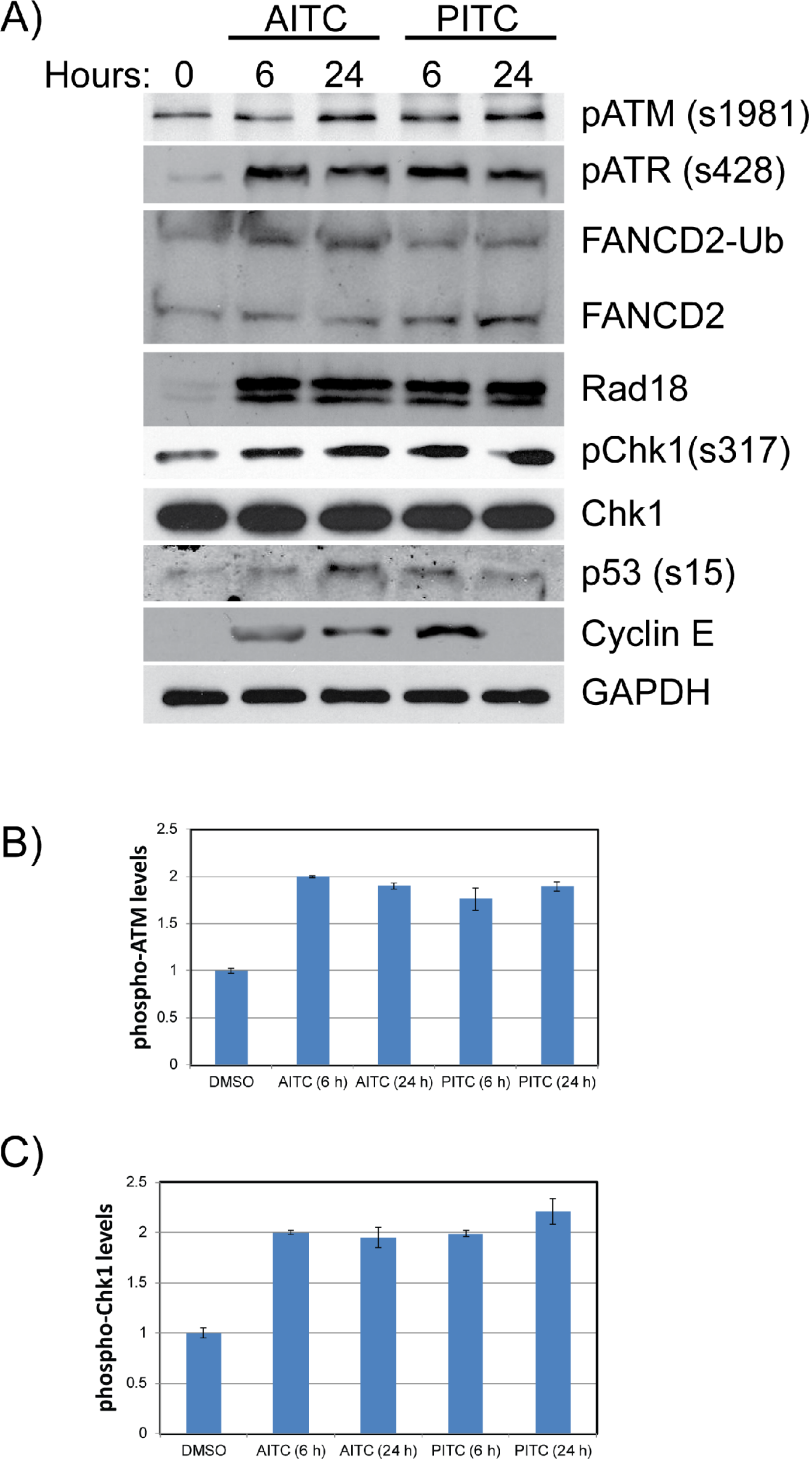
AITC exposure induces replication associated DNA damage and activates cell cycle checkpoints in A549 cells Exponentially growing A549 cells **(A)** were exposed to 20 μM AITC or PITC and cell lysates were prepared after indicated times. The normalized proteins were resolved on SDS-PAGE and blotted for different DDR proteins. Quantitation of p-ATM **(B)** and pChk1 **(C)** proteins are shown as bar diagram. Data presented are an average values from three independent experiments and ± SD presented as error bars.

**Figure 5:**
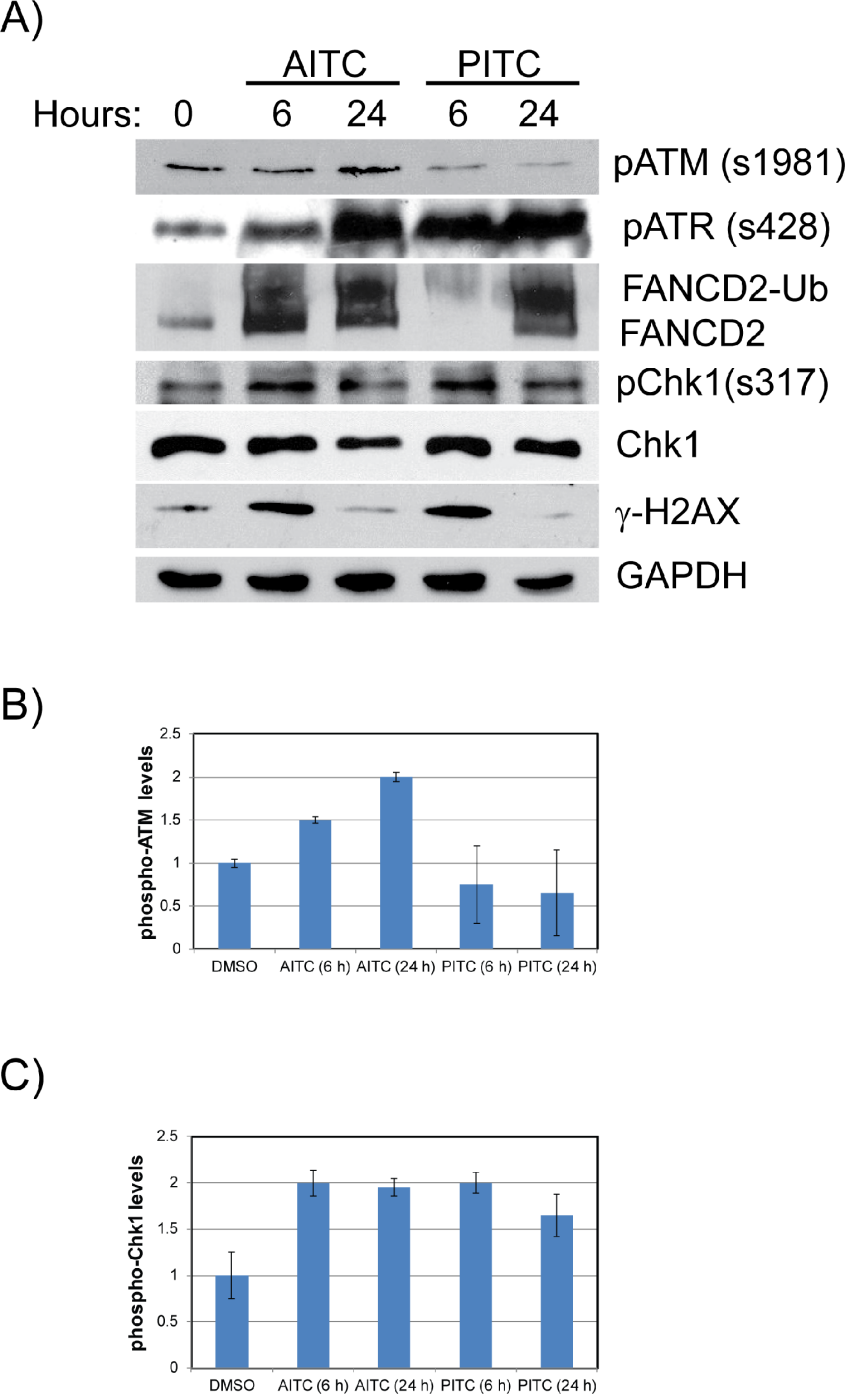
AITC exposure induces replication associated DNA damage and activates cell cycle checkpoints in H1299 cells Exponentially growing H1299 cells were exposed to either 20 μM AITC or 20 μM PITC and cell lysates were prepared after 6 and 24 hours of drug treatment. The normalized proteins were resolved on SDS-PAGE and blotted for different DDR proteins **(A)**. Quantitation of p-ATM **(B)** and pChk1 **(C)** proteins are shown as bar diagram. Data presented are an average of three independent experiments and ± SD presented as error bars.

Replication stress is known to induce DNA damage due to the stalled or collapsed forks, which then activates ATM/ATR-mediated cell cycle checkpoint responses to promote fork stability and restart thorough Rad18 and Fanconi anemia (FA) DNA repair pathways (monoubiquitinated FANCD2) [26]. To test whether ITCs also induce replication stress-associated DDR, A549 and H1299 cells were exposed to 20 μM AITC or PITC. After the indicated times of exposure (6 and 24 hours), whole cell lysates were normalized for protein concentrations and probed for different DDR proteins. Consistent with the cell cycle and immunofluorescence data, NSCLC cells treated with the AITC and PITC induced ATM/ATR-mediated DDR as evidenced by phosphorylation of ATM, ATR, p53 and Chk1 (Figures 4A–4C and 5A–5C), and induced the expression of replication stress-associated DNA repair proteins such as Rad18 (Figures 4A), mono-ubiquitinated FANCD2 (Figures 3 and 4A) and γH2AX (Figures 3, 5A and S3). Consistent with the differences observed in the cell survival and cell cycle data, H1299 cells treated with PITC exhibited reduced phosphorylated ATM compared to A549 cells (Figure 5A and 5B). However, the persistence of phosphorylated ATR after 24 hour drug treatment indicates the activated DDR in these cells, which might contribute to slow progression through cell cycle (Figure 2, S1A and S2B), DNA repair (Figures 3, 4 and 5) and cell death pathways (Figure 7, Figure S2A). However, careful evaluation of replication dynamics in the context of individual ITC exposure and DNA repair events would be important to give more detailed information of their cellular effects. Similar to the cell cycle profiles (Figure 2 and S1), expression levels of cyclin E and cyclin B correlated in response to both the ITCs at 6 and 24 hours (Figure 4A and S1B).

### AITC inhibits migration of NSCLC cells

**Figure 6:**
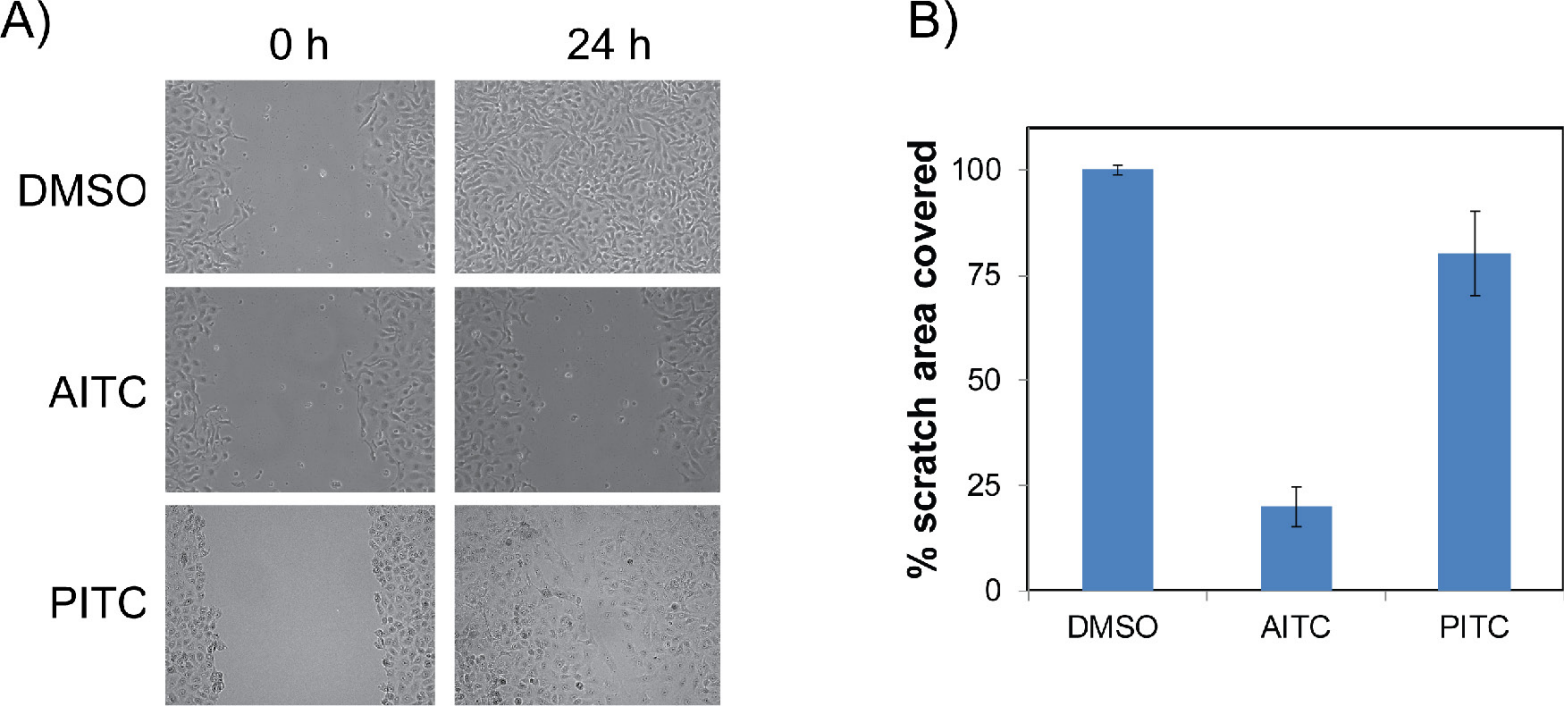
AITC is a potent inhibitor of NSCLC cells cell migration A549 cells were used for scratch assay and cell migration was measured for 24 hours after exposed to 10 μM AITC or PITC for 24 hours. Images of the scratch assays are shown before and after treatment with the ITCs treatments **(A)** The percent of cell migration quantified from three independent experiments. The average values were presented in the histogram and the error bars indicates ± SD **(B)**.

To assess whether AITC also affects cell migration, which is an indication of EMT and aggressive behavior of malignant disease, we performed scratch assays or wound healing assay using A549 cells and measured the cell migration by time lapse images up to 24 hours. As shown in Figures 6A and 6B, AITC significantly inhibited migration of A549 cells following 24 hours of treatment. The effect of PITC on cell migration was minimal compared to AITC at the concentrations used in this study (20 μM). The percentage of migration area covered after 24 hrs was almost 100% for DMSO treated control cells, while 21.1% and 80.9% for the cells treated with AITC and PITC respectively. We also observed that the rate of wound healing was faster in PITC treated cells compared to the cells treated with AITC. These results clearly indicate that the percentage of migration area of the AITC treated cells was significantly lower than that of the control cells and PITC treated cells (Figure 5B). These data suggests that AITC might inhibit metastatic potential of NSCLC cells.

### AITC induces apoptosis in NSCLC cells

**Figure 7:**
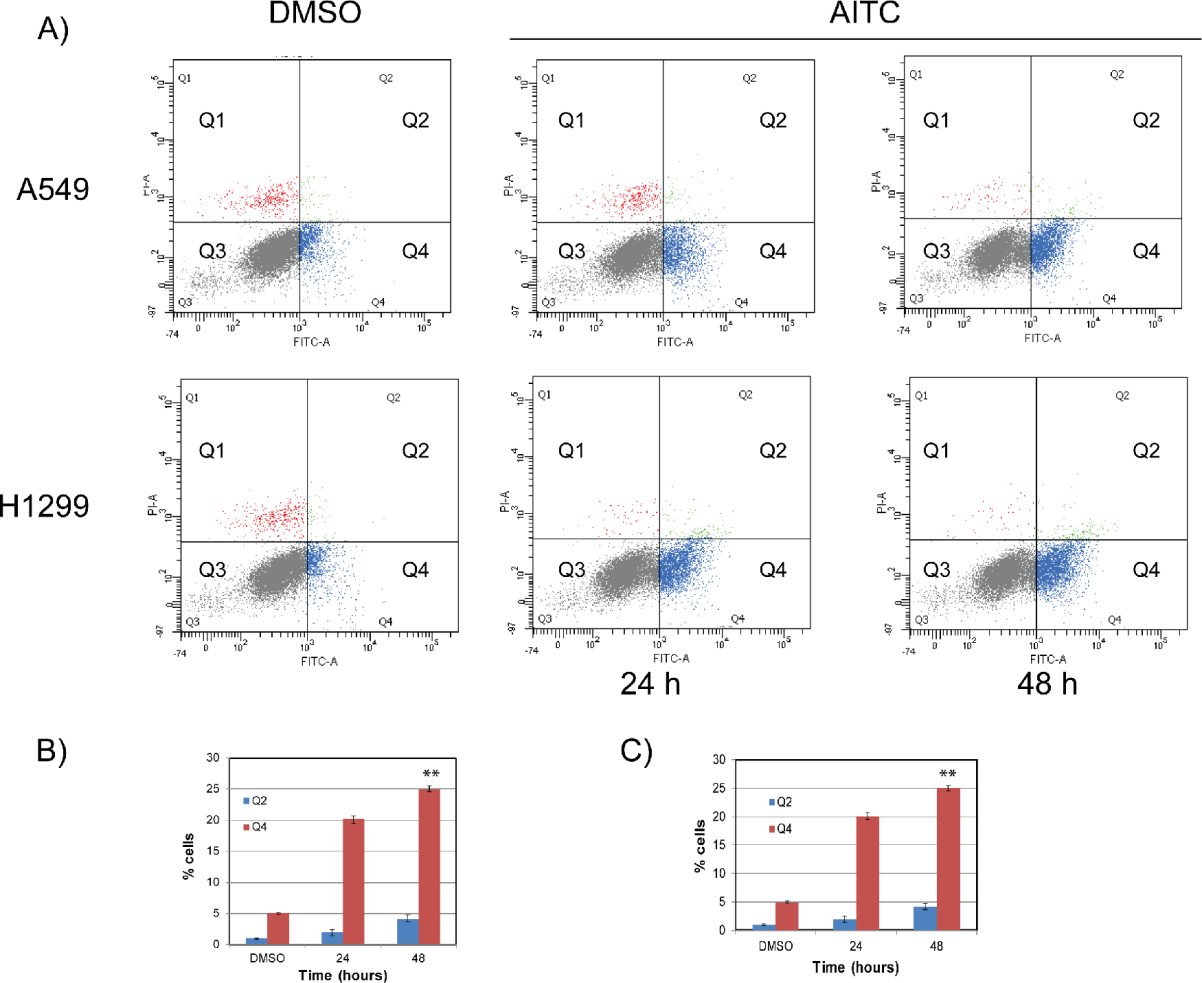
AITC-induces apoptosis in NSCLC cells A549 (top panel) and H1299 (bottom panel) cells were exposed to AITC for 24 or 48 hours and cells were co-stained with PI and Annexin V antibody and analyzed by flow cytometry. The data presented in (B) and (C) are the average values of three independent experiments for 24 and 48 hours respectively. The error bars represents ± SD (**P < 0.001).

To assess whether AITC-induced replication associated DDR and G2/M cell cycle arrest leads to apoptosis in NSCLC cells, we measured percent of cells undergoing apoptosis using annexin-V staining followed by flow cytometry analysis. First, we evaluated the concentration dependent effects of AITC on cell cycle and proapoptotic markers after 24 hours exposure. These results clearly demonstrated concentration dependent increase in proapoptotic proteins (Figure S2A) and cell cycle arrest in A549 cells (Figure S2B). To further evaluate A549 and H1299 cells were exposed to AITC and the cells undergoing apoptosis were assessed after 24 and 48 hours post treatment. As shown in the Figure 7A (top panel), AITC treatment induced about a 3 and 4 fold increase in annexin-V positive cells at 24 and 48 hours in A549 cells (Figure 7B), respectively. Similar results were observed in H1299 cells treated with AITC (Figures 7A bottom panel and 7C).

### AITC exhibits synergistic therapeutic effects on NSCLC cell lines in combination with radiation

**Figure 8:**
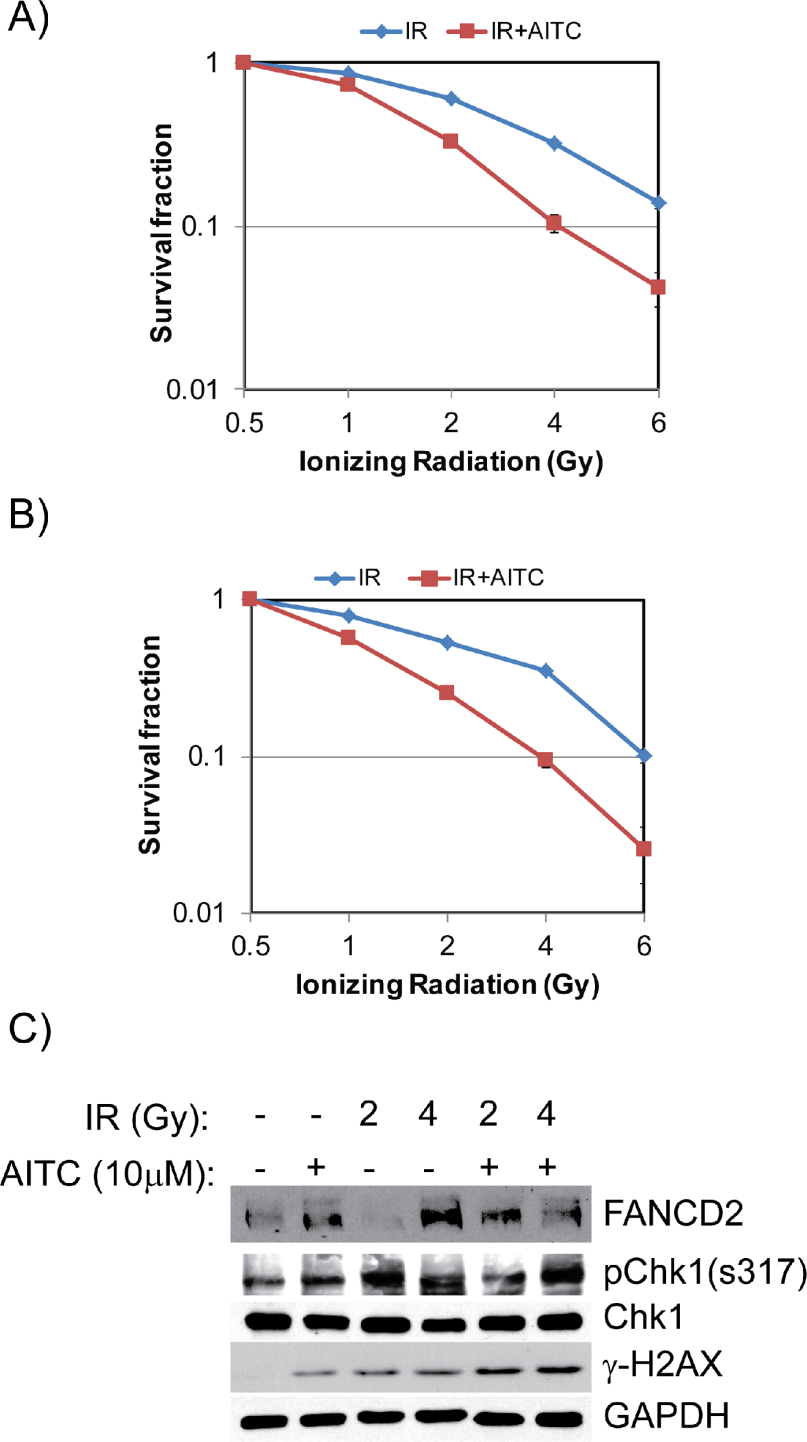
AITC pretreatment sensitizes NSCLC cells to radiation treatment Clonogenic survival assays were performed after pretreating A549 and H1299 cells with 5 μM AITC and exposed them to different doses of ionizing radiation. After 10 days colonies were counted and plotted as percent survival fraction for A549 **(A)** and H1299 cells **(B)**. Pretreatment with AITC enhances radiation induced DDR in A549 cells **(C)**. The data presented in **(A)** and **(B)** are mean of three independent experiments and the error bars indicates ± SD

**Figure 9:**
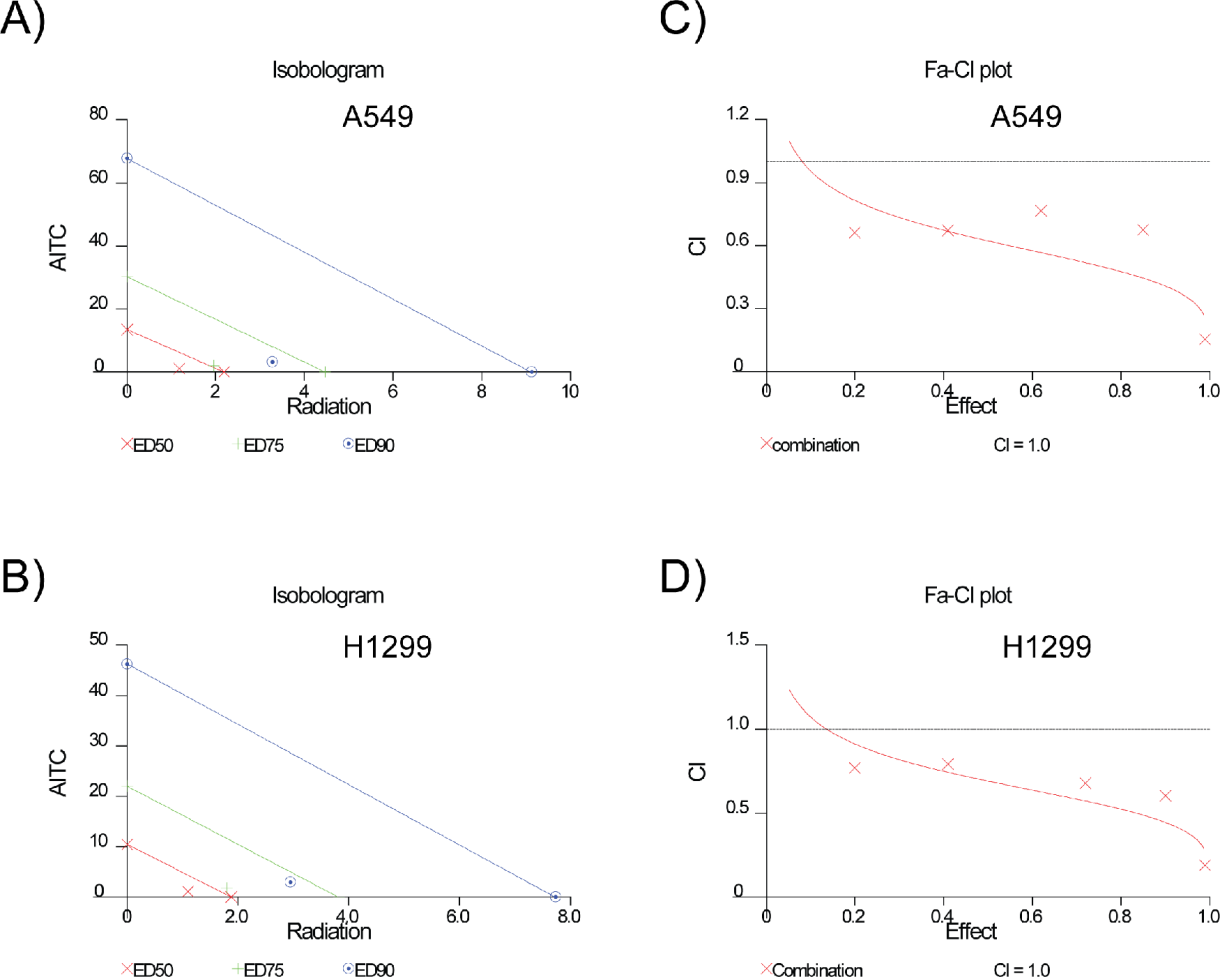
AITC and radiation combination therapy exhibits synergistic therapeutic activity in NSCLC cells The Isobologram curve showing the synergistic effect of AITC with Radiation in A549 cell line **(A)** and H1299 cell lines **(B)** at different effective doses (ED). The combination index plots for AITC and radiation combination therapy shows synergy all the Fractions affected (Fa) for both A549 **(C)** and H1299 **(D)** cells. Considering CI > 1, antagonistic; C*I* = 1, additive and CI < 1, synergistic.

It is evident from several studies that agents that arrest cells in S and G2/M phases work synergistically with radiation therapy, an important treatment regimen used for local and advanced lung cancer [27]. Radiation therapy becomes the primary option for lung cancer patients, whose lung cancers are limited to the chest but cannot be resected surgically. In order to study the effect of AITC treatment in combination with radiation, we determined the most effective doses for combining the two agents. Briefly, A549 and H1299 cells were pretreated with a fixed concentration of AITC for overnight and exposed to varying doses (0.5Gy to 6Gy) of radiation and allowed to form colonies. The survival fraction of the cells were measured by counting the colonies (>25 cells) after 10 days. The impact of AITC and radiation-induced cytotoxicity is depicted in Figures 8A and 8B. The combination of AITC and radiation indicated increased cytotoxicity compared to the single agents (radiation alone or AITC treatment alone) against NSCLC cells. Consistent with the survival data, combination treatment also induced increased γH2AX and phosphorylated Chk1 in these cells (Figure 8C). Based on these results, we hypothesized that AITC may have synergistic effect on radiation therapy. To further evaluate the combination therapy, a fixed AITC and radiation ratio was selected (AITC:IR) and used in further experiments. From these values, combination index (CI) values were calculated to quantitatively measure the potential for additive, synergistic, or antagonistic interactions. To further evaluate the combination therapy of AITC and radiation, the percent of survival fraction by either agent alone was quantified comparing to untreated cells. Similarly, reduction in survival fraction by combination of AITC and radiation treatment was also calculated. From these values, combination index (CI) values were calculated using CalcuSyn software. As shown in the isobolograms for A549 and H1299 (Figures 9A and 9B respectively), combination of AITC pre-treatment with radiation resulted in more-than-additive cell killing in both of the NSCLC cell lines. Since this suggested synergistic cell killing, we next examined the ability of AITC to synergize with radiation using the Chou-Talalay synergy analysis method as described previously [28]. The isobolograms were drawn for these values, representing 50%, 75% and 90% growth inhibition (ED) for both A549 and H1299 cells (Figures 9A and 9B). As indication by CI plots (Figures 9C and 9D), AITC and radiation combination treatment synergistically killed both the NSCLC cell lines at most fractions affected (Fa). The CI values for the fraction affected (Fa) at ED50, ED75 and ED90 are all < 1 for both A549 and H1299 cell lines (Table 1). These results indicate that AITC could be a potential radiation sensitizing agent for the treatment of NSCLC.

**Table 1:** The combination indexes for A549 and H1299 NSCLC cells treated with different effective doses (ED) of AITC and radiation combinations The combination-index values are depiction of a pharmacological interaction of two drugs. A CI = 1 indicates an additive effect between the two agents, whereas a CI < 1 indicates, synergism while CI > 1 indicates antagonism

Cell line	ED50	ED75	ED90
A549	0.69413	0.55769	0.44815
H1299	0.62315	0.50414	0.40826

To further evaluate these synergistic cytotoxic effects of AITC and radiation, and if combination therapy is due to increased DNA damage, we further evaluated DDR markers. A549 cells were treated with either 10 μM AITC or DMSO and exposed to different doses of IR. Consistent with the survival data, combined treatment of AITC and radiation elicited increased levels of DDR, as evidenced by increased levels of γH2AX, FANCD2 and pChk1 proteins compared to control and individual agents treated cells. These data suggest that AITC pre-treatment in combination with radiation therapy may result in a more pronounced therapeutic activity in NSCLC.

## DISCUSSION

AITC is a naturally occurring isothiocynate, which is abundant in many cruciferous vegetables that have been extensively evaluated for their chemopreventive properties in several cancer models [29]. However, the mechanism for the ITC-induced antitumor activities is not well defined and numerous pathways have been implicated. Studies on many tumor cell models have demonstrated that their antineoplastic effects are at least partly due to the G2/M cell cycle arrest and mitochondria-mediated apoptosis [29, 30, 31, 32]. It is evident from several investigations that ITCs cause change in redox potential, inhibit cellular enzymes such as DNA topoisomerases, tubulins and affect regulation of several genes in tumor cell survival and growth [18, 21–24, 30]. Recently, several studies also indicated that AITC and related ITCs induces DNA damage and cell cycle arrest in tumor cells [18, 21, 22, 32, 33]. However, these agents are highly reactive and may influence the function and stability of many proteins in the cells, which makes it difficult to predict the specific cellular targets that are responsible for inducing DNA damage [23, 31, 32]. In this regard, we primarily focused our studies on DNA damage mediated effects of AITC on NSCLC cells and extended this knowledge in the context of cancer therapeutics. In our studies, naturally occurring AITC and the synthetic PITC both showed chemotherapeutic properties against NSCLC cells in a concentration-dependent manner. Interestingly, our results demonstrated that AITC interfere with cell cycle progression by inducing replication-associated DNA damage, as evidenced by γH2AX and FANCD2 (Figures 3, 4, 5 and S3). Consistent with the effects of S-phase poison CPT (Figure S1B and S1C), exposure of cells to AITC activated ATM/ATR mediated cell cycle checkpoint responses that attenuated NSCLC cells' progression through S-phase leading to their accumulation in G2/M phase. Importantly, most of the AITC-induced FANCD2 nuclear foci co-localized with BrdU foci, a nucleoside analogue and marker of active replication. Moreover, the appearance of DDR signals within 6 hours of ITCs exposure implies that these agents are potent DNA damage inducing agents. Together, these data suggests that AITC induces replication associated DNA damage in NSCLC cells, which might be a possible reason that their cytotoxic effects are specific to tumor cells [33, 34].

It is evident from several studies that the chemotherapeutic activities of various ITCs are highly variable based on the tumor model studied and in some cases different cell lines within the same tumor model [23, 30, 32, 35, 38]. Though both AITC and PITC induced replication associated DDR in NSCLC cells, PITC was less effective in inducing cell cycle arrest and other growth inhibitory properties. Similar results were observed previously using PITC in prostate cancer cells when compared with naturally occurring phenethyl isothiocyanate (PEITC) [31].

Fanconi anemia DNA repair pathway plays an important role in maintaining genome integrity by closely associating with replication machinery to stabilize stalled replication forks and facilitating the repair of collapsed forks by homologous recombination mediated repair [39]. We and others have previously shown that agents which perturb DNA replication or induce replication stress can activate the FA pathway, resulting in mono-ubiquitination of FANCD2 and its nuclear foci formation [25, 26, 37, 39, 40]. Consistent with these studies, AITC exposure activated FA pathway, replication-associated DDR, and induced cell cycle arrest and apoptosis. These results indicate that AITC can induce potentially lethal S-phase specific DNA lesions such as those induced by S-phase specific poison camptothecin [36, 42, 44, 45, 48]. Consistent with the camptothecin induced cellular responses (Figure S1B and S1C) and as described [42], inhibition of replication by aphidicolin markedly reduced AITC-induced cytotoxicity (Figure S4). Recently, Geng and colleagues reported similar observations, where hydroxy urea pretreatment abrogated AITC-induced apoptosis in human bladder cancer cells [43]. Together, these observations suggest that ITCs-induced DDR closely resemble to the S-phase specific poison camptothecin-induced DDR, a DNA topoisomerase 1 targeting anticancer agent (Figure S1B and S1C) [26, 42, 45].

Radiation therapy is a very common curative therapy for treatment of lung cancer, especially for patients whose lung cancers are limited to the chest but cannot be removed surgically. Though the mechanisms of radiation sensitizers are not fully known, it is plausible that they possess this activity by enhancing the damage induced by radiation or modulating the cell cycle distribution into the phases that are more vulnerable to radiation [45–48]. Previous studies demonstrated that cells in G0 or G1 are less sensitive to ionizing radiation [45], indicating agents that can alter the distribution of the cells into S and G2/M phases could be more susceptible to radiation [27]. This has been proven in many tumor models including NSCLC cancer using DNA topoisomerase 1 (Top1) poisons such as camptothecin (CPT) [45, 46, 48]. The radiation sensitization properties of these agents were attributed to induction of low dose DNA damage in replicating cells that slows the cells cycle progression through S-phase and arrest them in G2/M phase. This was further confirmed because only pre-treated cells but not the post-treated cells exhibited radiation sensitivity [48].

Since dietary AITC demonstrated replication-associated DDR and cell cycle arrest in S and G2/M phases similar to those observed in response to topoisomerase-1 inhibitors, we further assessed whether these agents could be radiosensitizers in NSCLC cells [46]. As hypothesized, pretreatment of cells with a sub-lethal dose (5 μM) of AITC, remarkably hyper-sensitized A549 and H1299 cells compared to radiation treatment alone. The radiosensitizing effect of AITC was very significant in exhibiting synergic interactions as indicated in the Figure 9 and Table 1. As AITC is a dietary constituent of many vegetables and highly bioavailable, its therapeutic use may be well tolerated compared to other chemotherapeutic drugs. Moreover, several studies in animal models demonstrated AITC causes minimal adverse effects at much higher doses then the concentrations used in this study [49, 50]. In summary, these data provide compelling evidence that dietary ITCs such as AITC can induce replication stress in NSCLC cells by generating fork-stalling DNA lesions. Importantly, these data establish a strong basis for the preclinical evaluation of AITC and other dietary isothiocyanates in combination with radiation therapy in treatment of NSCLC. Since radiation therapy remains the common treatment method for standard of care therapy for NSCLC, we propose that AITC and other dietary isothiocyanates may provide important therapeutic effects, in combination with radiation to eliminate locally advanced and refractory NSCLC tumors.

## METHODS

### Cell lines and antibody

The NSCLC cell lines A549 (human adenocarcinoma epithelial cell line) and H1299 (human were cultured in Dulbecco's modified Eagle's medium, supplemented with 10% FBS, 100 μg/ml streptomycin sulfate and 100 U/ml penicillin. Normal human bronchial epithelial cells were grown in BEGM™ Bronchial Epithelial Cell Growth Medium as described previously [51]. Cells were routinely tested for mycoplasma contamination using Mycotest kit (Invitrogen) and cells within 10 passages were used in the experiments. AITC and PITC (Sigma, St. Louis, MO) stock solutions were prepared by dissolving in anhydrous DMSO and stored at −20°C. These stock solutions were further diluted to required concentration before adding to the cells. Antibodies to the following antigens used in this study include: ATR, ATM, Chk1, FANCD2 and GAPDH were from Santa Cruz Biotechnology, Inc.; Rad18, from Bethyl Laboratories, Inc.; phospho-ATM-Ser1981, phospho-Chk1 Ser-317 were Cell Signaling Technologies, γH2AX is from Millipore. The secondary antibodies like anti-mouse IgG-Cy3, anti-mouse IgG-FITC, anti-rabbit IgG-FITC were from Molecular Probes.

### Clonogenic survival assays

Cells were plated in 6-well dishes in triplicates, allowed to attach for 16 hours and treated with indicated concentrations of the therapeutic agents and allowed them to form colonies by replacing medium every three days. After 7 to 12 days colonies were fixed in methanol, stained with crystal violet and the colonies having more than 25 cells were counted using Gene Tools, Syngene Imaging system [52].

### Cytotoxicity assays

Cells were counted and approximately 300 cells were seeded in 6 well plates and allowed to attach overnight. These cells were treated with indicated concentrations of ITCs and control cells received DMSO. After three days of incubation cells were trypsinized and counted using Tryphan blue exclusion assay (Invitrogen).

### Western blotting

Cells were exposed to the indicated agents and proteins from whole cell lysates were prepared after washing the cells with ice cold PBS. Cells were lysed in ice-cold cytoskeletal (CSK) buffer (10 mM PIPES (pH 6.8), 100 mM NaCl, 300 mM sucrose, 3 mM MgCl2, 1 mM EGTA, 1 mM dithiothreitol, 0.1 mM ATP, 1 mM Na3VO4, 10 mM NaF and 0.1% Triton X-100) freshly supplemented with protease and phosphatase inhibitors (Roche). After normalizing the protein concentrations, samples were prepared in 4x SDS-PAGE sample buffer and heated to 100°C for 15 min. Denatured samples were resolved by SDS-PAGE and transferred them to nitrocellulose membranes. Membranes were incubated with indicated antibodies followed by respective HRP-conjugated secondary antibodies and blots were developed by chemiluminscense detection kits.

### Immunofluorescence

Cells were seeded in to 35 mm glass bottom dishes (FlouroDish). The resulting cells were treated with AITC or PITC (or DMSO for controls). Cells were fixed in 3% formaldehyde for 10 min and then in 100% methanol (−20°C) for 10 min at room temperature. Fixed cells were blocked in 10% FBS for 30 min. After three washes with PBS, cells were incubated overnight at 4°C with primary antibodies in PBS containing 5% bovine serum albumin (BSA) and 0.1% Triton X-100 (PBS-T). The slides were washed three times with PBST containing 1% BSA then incubated with fluorescently-labeled secondary antibodies for 2 hours at room temperature.

### Irradiation

Cells were plated in 6 cm plate and next day treated with AITC for 16 hours. After drug treatment cells were exposed to X-rays using a particle linear accelerator, producing X photons of 10 MV at a dose rate of 0.5 Gy/min to 6 Gy/min (X-rad Precision X-ray). The irradiator was at a fixed distance from the target, and the irradiation field was about 40 × 40 cm. The 6 cm plates were always placed in the center of the field.

### Cell cycle analysis by flow cytometry

After 6 hours and 24 hours of exposure with DMSO, AITC and PITC, cells were harvested by fixing in ice cold 70% ethanol and cell cycle profiles were analyzed by flow cytometry after propidium iodide (PI) staining as described previously [52].

### Apoptosis assay

Apoptosis assays were performed by treating the cells with AITC or PITC for 24 hours and 48 hours. Cells undergoing apoptosis were measured after labeling with PE-annexin-V apoptosis detection kit (Dead Cell Apoptosis Kit with Annexin V Alexa Fluor^®^ 488 & PI according to the manufacturer instruction (Life technologies Inc.) and analyzed by flow cytometry (BD Bioscience) [53].

### Drug and radiation synergy analysis

AITC and Radiation synergy was determined by using the combination-index methods and isobologram, derived from the median effect principle of Chou and Talalay [28], using the CalcuSyn software 2.1 (Biosoft, UK). Data obtained from the cell survival assays was used to perform these analyses. The isobologram method is a graphical demonstration of the pharmacologic interaction of two drugs, and a desired fractional affected (Fa). In isobologram straight line connect the Fa points against experimentally used fixed ratio combinations of radiation and the AITC on X- and Y-axes to generate isobolograms. The combination data points that reside on the line represent an additive interaction while data points that were below and above the line represent synergism and antagonism respectively. The combination-index is a mathematical and quantitative depiction of a pharmacological interaction of two drugs. A CI = 1 indicative of an additive effect between the two agents, whereas a CI < 1 indicates, synergism while CI > 1 indicates antagonism.

### Statistical analysis

The data presented in clonogenic survival assays are representative of three independent experiments performed in triplicates at each time. Error bars represents the ± standard deviation. Data were analyzed using GraphPad Prism 6 and Microsoft Excel. All other data presented in the manuscript repeated at least 2 times to confirm the reproducibility of the results.

## SUPPLEMENTARY FIGURES


