# Supplementary material for: Allyl isothiocyanate induces replication-associated DNA damage response in NSCLC cells and sensitizes to ionizing radiation
Source: Oncotarget. 2015 Jan 27;6(7):5237–52. doi: 10.18632/oncotarget.3026 (PMC4467145; doi:10.18632/oncotarget.3026)
Supplement: Supplementary file 1 [file oncotarget-06-5237-s001.pdf]

SUPPLEMENTARY FIGURES

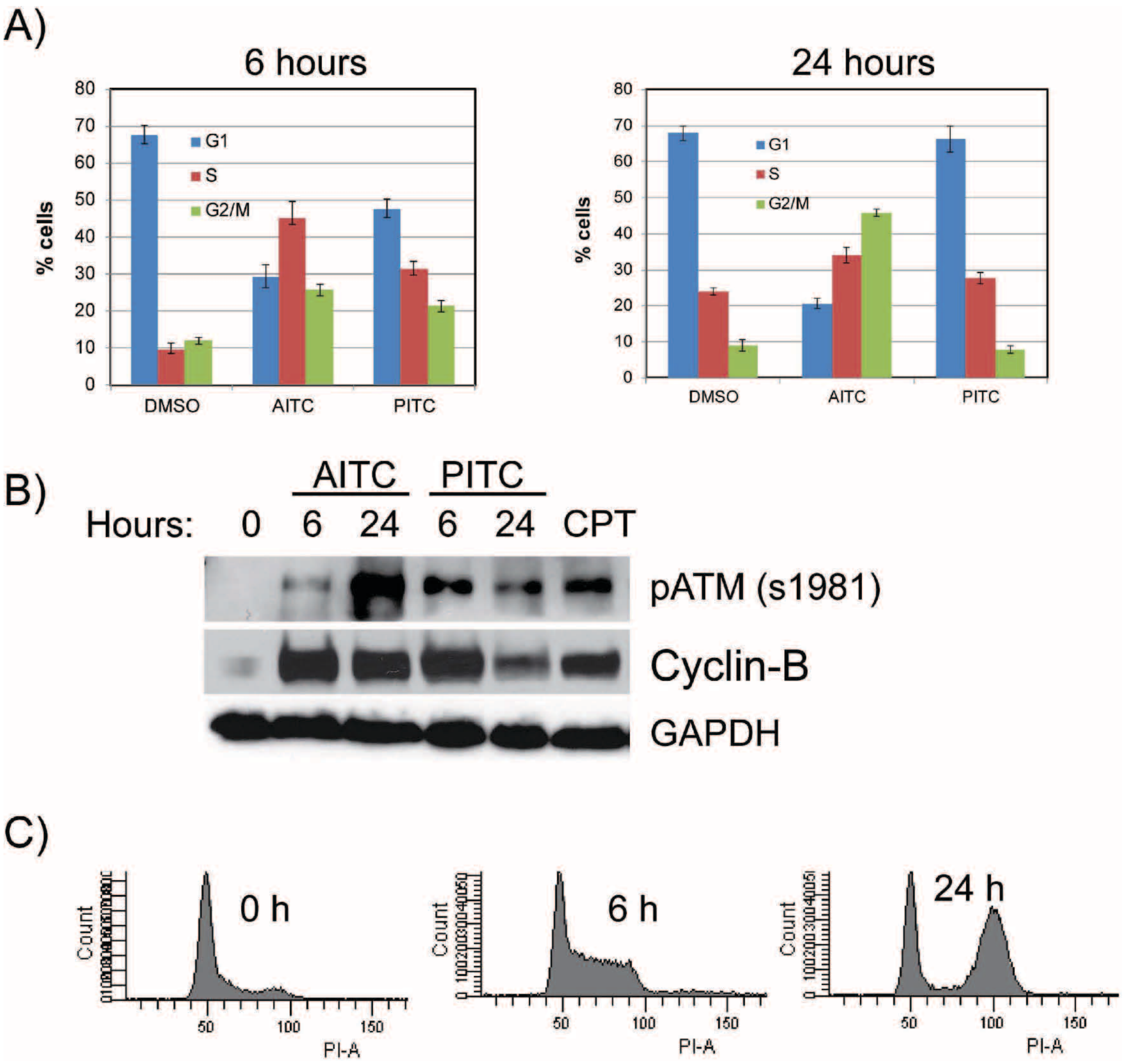

Supplementary Figure S1: AITC exposure induces similar DDR (B) and cell cycle profiles as S-phase specific poison camptothecin (CPT). Cell cycle profiles of A549 cells exposed to AITC after 6 and 24 hours **(A).** AITC exposure induces phosphorylation of ATM and induces G2 arrest as indicated by elevated levels of cyclin B **(B).** Cell cycle profiles of S-phase specific poison CPT (50 nM) indicates S and G2 arrest at 6 and 24 hours exposure times respectively **(C).** Datapresented in (A) are average of three independent experiments and error bars indicates +/− SD.

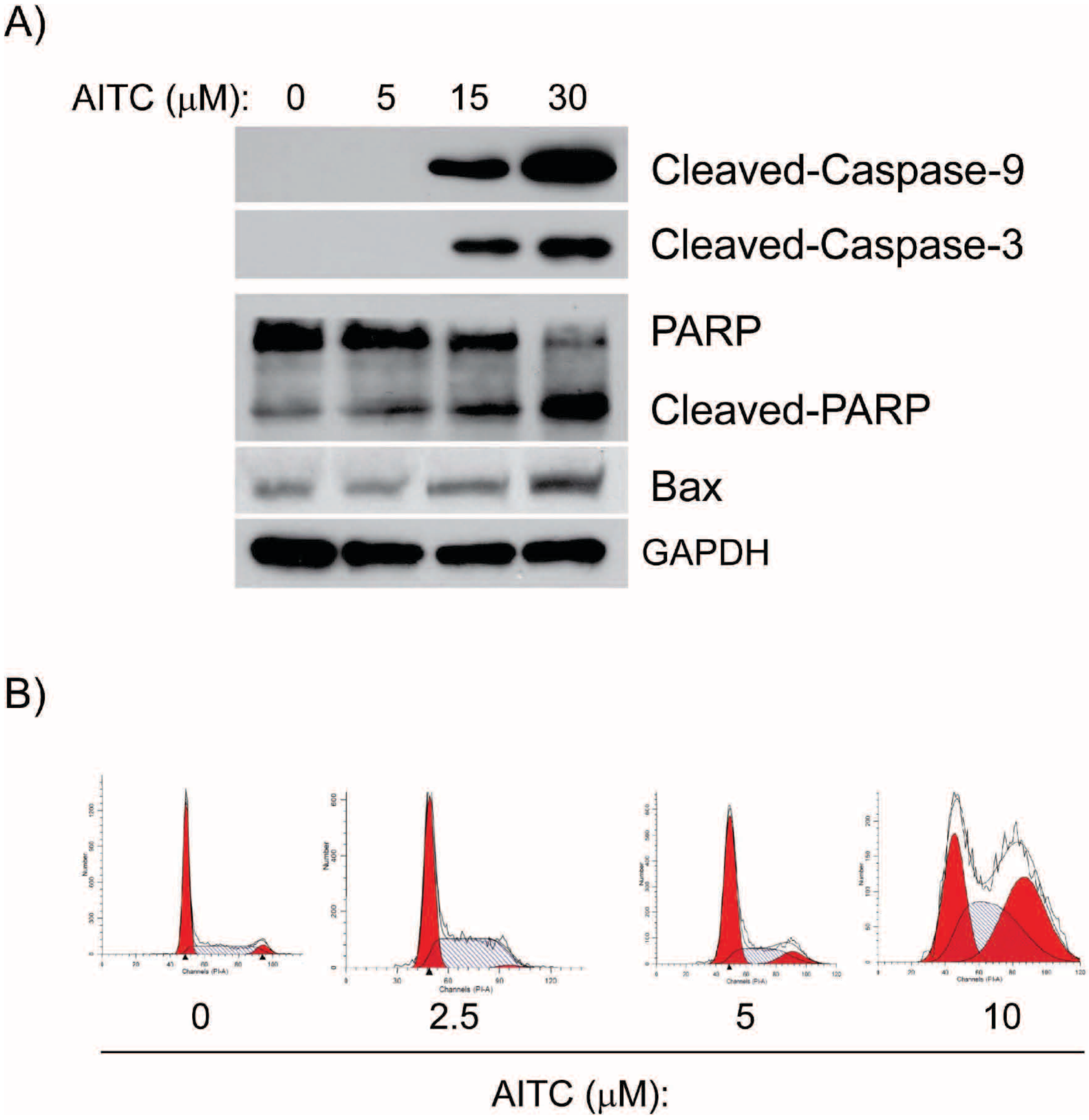

Supplementary Figure S2: AITC induces concentration dependent cell cycle arrest in S/G2 phases and apoptosis in NSCLC cells. A549 cells were exposed to indicated concentrations of AITC for 24 hours and proteins from whole cell lysates were immuno blotted for indicated proteins **(A).** A549 cells were exposed to indicated concentrations of AITC for 24 hours and cell cycle profiles were assessed using flow cytometry **(B)**.

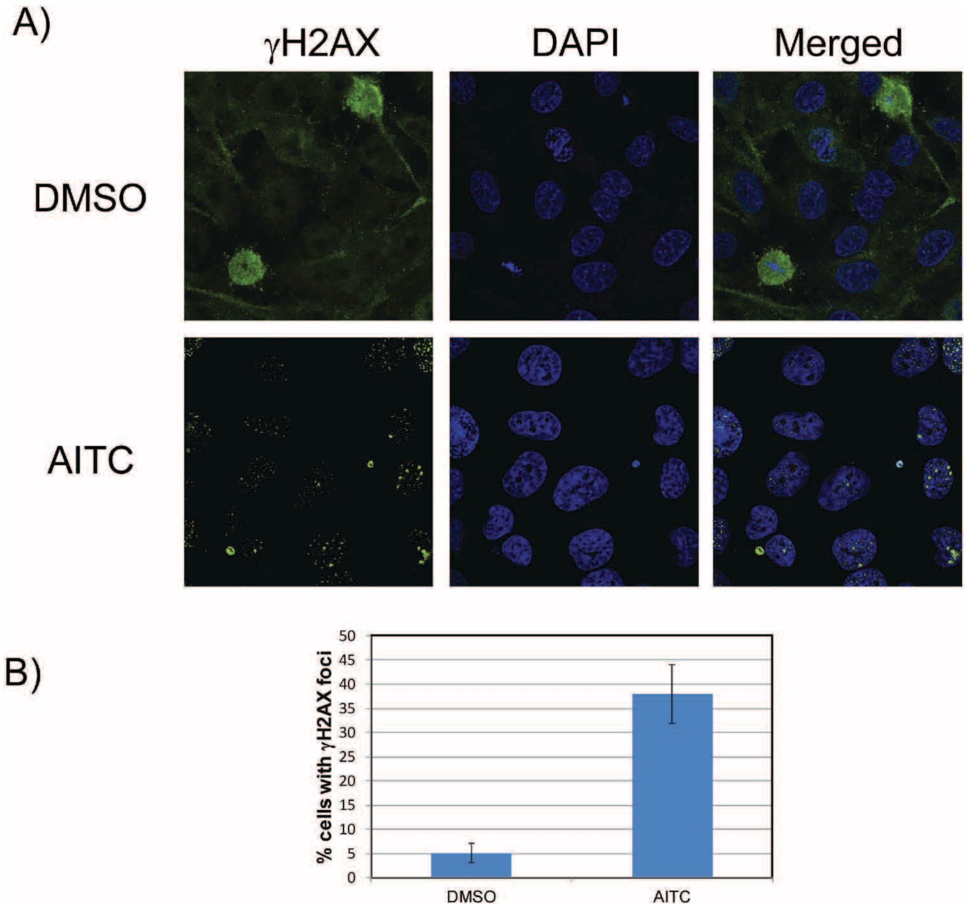

Supplementary Figure S3: AITC induces γH2AX foci in A549 cells. A549 cells were exposed to 20 µM AITC for 6 hours and the cells were fixed and immuno stained with γH2AX antibody followed by FITC-conjugated secondary antibody. A representative image showing γH2AX foci positive cells in AITC treated cells **(A).** Cells positive for γH2AX foci were enumerated in at least 20 focal images and average values presented as histogram **(B).** Error bars indicates +/− SD.

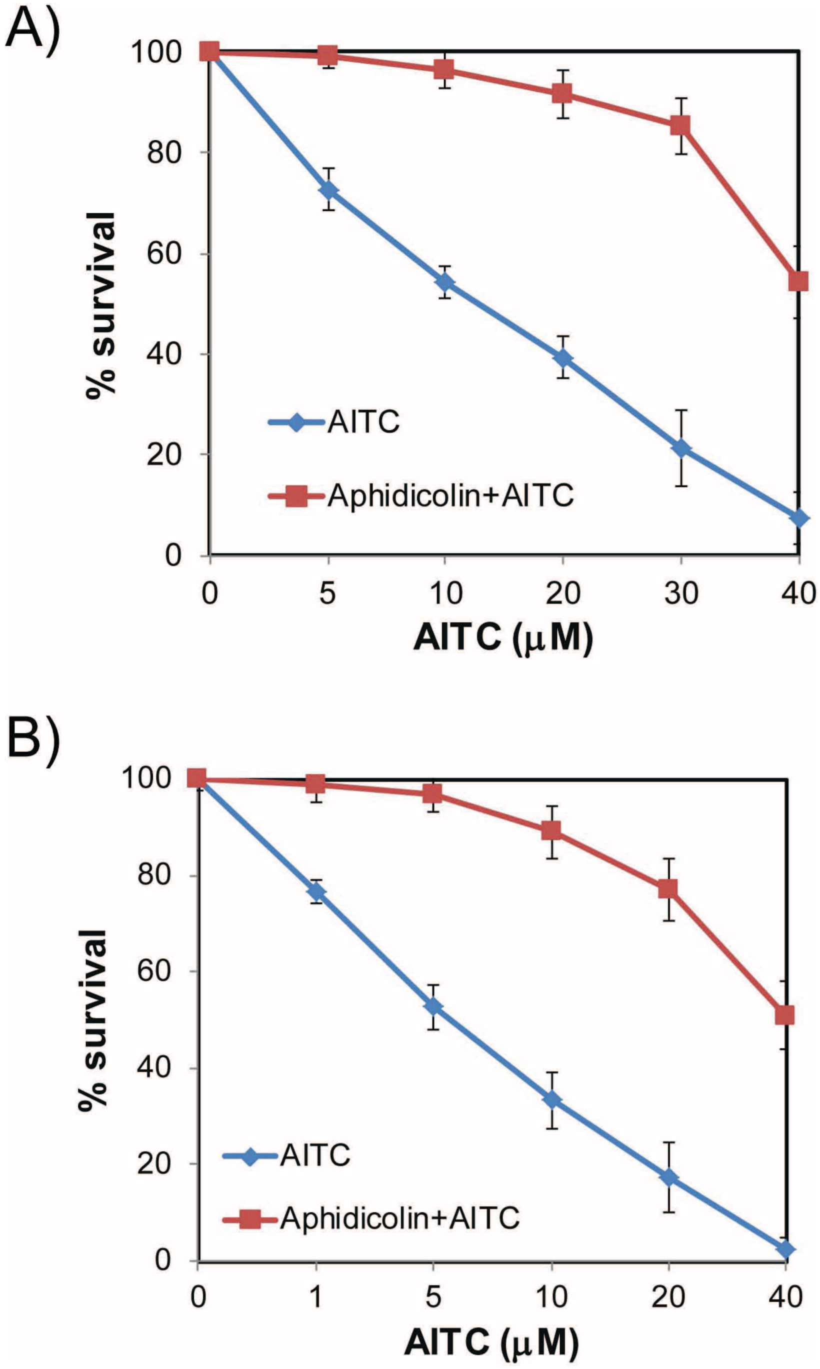

Supplementary Figure S4: Aphidicolin inhibits AITC-induced cytotoxicity in NSCLC cells. Exponentially growing A549 **(A)** and H1299 **(B)** cells were pretreated for 6 hours with 10 μM Aphidicolin or DMSO and then exposed to indicated concentrations of AITC. After 78 hours cell viabilities were assessed using CellTitre Glow luminescent assay kit (Promega). Data presented were average values of triplicates and the error bars indicates +/– SD.

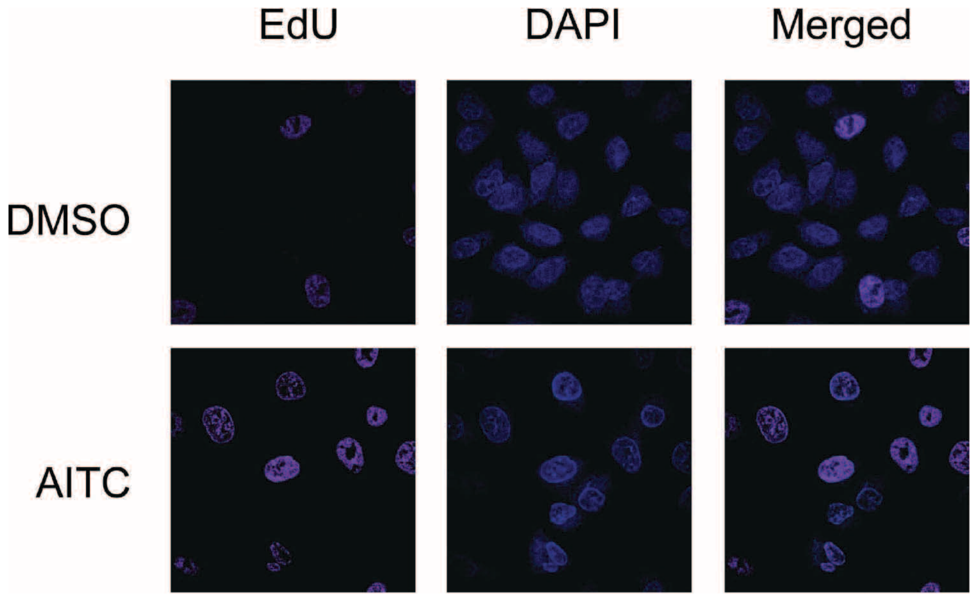

Supplementary Figure S5: AITC exposure transiently accumulates cells in S-Phase. H1299 cells were exposed to 20 µM AITC or DMSO for 6 hours and the cells were labeled with EdU (5-ethynyl-2'-deoxyuridine) for 20 minutes before fixing. Methanol fixed cells were fluorescently labled using Click-iT assay kit (Life technologies) and images were processed. A representative image showing EdU foci positive cells, a marker of S-phase (replicating cells) for both DMSO and AITC treated cells.
